# ELDER FAMILY FINANCIAL EXPLOITATION BY POWER-OF-ATTORNEY AGENTS THROUGH THE LENS OF BRONFENBRENNER’S PPCT MODEL

**DOI:** 10.1093/geroni/igz038.1410

**Published:** 2019-11-08

**Authors:** Virginia B Vincenti, Ashton Chapman

**Affiliations:** 1 University of Wyoming, Laramie, Wyoming, United States; 2 Social Grove, Inc., Joplin, Missouri, United States

## Abstract

This paper presents a subset of qualitative data from a phenomenological study of 3 men and 17 women (N=20) from families with designated power of attorney (POA) agents who allegedly perpetrated elder family financial exploitation (EFFE). Participants were aged 22 to 63 (M = 43.5) with varying educational and income levels. The study explored EFFE by POA within Bronfenbrenner’s PPCT model. Data were analyzed using a thematic, inductive approach. Person characteristics (e.g. perpetrators’ personality and victims’ cognitive functioning), proximal processes (e.g. family patterns of communication and resource sharing), context (e.g. geographic location), and time (e.g. prevailing legal, economic, and cohort factors) emerged as relevant for EFFE experiences. Given that EFFE helping professionals (e.g., attorneys, practitioners) often lack training in family- or systems-focused dynamics and interventions, implications and applications of the PPCT model will be discussed with the goal of raising awareness of factors related to EFFE identification and prevention.

